# Impact of post-implant dosimetric parameters on the quality of life of patients treated with low-dose rate brachytherapy for localised prostate cancer: results of a single-institution study

**DOI:** 10.1186/s13014-015-0434-4

**Published:** 2015-06-10

**Authors:** Antonello Veccia, Orazio Caffo, Giovanni Fellin, Salvatore Mussari, Francesco Ziglio, Francesca Maines, Luigi Tomio, Enzo Galligioni

**Affiliations:** Medical Oncology Unit, Santa Chiara Hospital, Largo Medaglie d’Oro 1, 38100 Trento, Italy; Radiotherapy Unit, Santa Chiara Hospital, Largo Medaglie d’Oro 1, 38100 Trento, Italy; Health Physics Unit, Santa Chiara Hospital, Largo Medaglie d’Oro 1, 38100 Trento, Italy

**Keywords:** Brachytherapy, Dosimetry, Prostate cancer, Quality of life

## Abstract

**Background:**

To assess the relationship between dosimetric parameters and the quality of life (QL) outcomes of patients with low-intermediate-risk localised prostate cancer (LPC) treated with low-dose-rate brachytherapy (LDR-BT).

**Materials and methods:**

We evaluated the participants in two consecutive prospective studies of the QL of patients treated with LDR-BT for LPC. QL was evaluated by means of a patient-completed questionnaire assessing non functional [physical (PHY) and psychological (PSY) well-being, physical autonomy (POW), social relationships (REL)] and functional scales [urinary (URI), rectal (REC), and sexual (SEX) function]; a scale for erectile function (ERE) was included in the second study. Urethra (D10 ≤ 210 Gy) and rectal wall constraints (V100 ≤ 0.5 cc) were used for pre-planning dosimetry and were assessed with post planning computerized tomography one month later for each patient.

**Results:**

QL was assessed in 251 LPC patients. Dosimetry did not influence the non-functional scales. As expected, a progressive impairment in sexual and erectile function was reported one month after LDR-BT, and became statistically significant after the third year. Rectal function significantly worsened after LDR-BT, but the differences progressively decreased after the 1-year assessment. Overall urinary function significantly worsened immediately after LDR-BT and then gradually improved over the next three years. Better outcomes were reported for V100 rectal wall volumes of ≤ 0.5 cc and D10 urethra values of ≤ 210 Gy.

**Conclusions:**

The findings of this study show that dosimetric parameters influence only functional QL outcomes while non-functional outcomes are only marginally influenced.

## Introduction

Low-dose-rate brachytherapy (LDR-BT) via a permanent implant is an established option for the radical treatment of localized prostate cancer (LPC) [1], and leads to at least the same disease control as other local treatments in low–and intermediate-risk patients [2, 3]. Furthermore, it is a treatment procedure that can be done as an out-patient procedure or maximal one overnight stay with a low burden of side effects and the possibility of preserving the patients’ quality of life (QL). The side effects are mainly related to the irradiation of the rectal wall and urethra, and are usually transient provided that the patients are adequately selected and specific dosimetric parameters are respected [4]. The impact of LDR-BT on the QL has been extensively assessed in a number of prospective studies; Van Gellekom et al. and Vordermark et al. demonstrated that most of the symptoms resolve within the first year after implantation and reach a steady state by three years [5, 6].

In our hospital, LPC patients treated with LDR-BT have been involved in two consecutive prospective studies aimed at assessing their QL: the first evaluated only patients treated with LDR-BT and the final results were published in 2006 [7]; the second (comparing the QL of patients treated with LDR-BT or radical prostatectomy) enrolled patients until July 2010 and is still in its long-term follow-up phase, although the preliminary results have been reported [8]. Despite the clear relationship between dosimetric parameters and the entity of side effects [9, 10], only a few studies have evaluated their impact on the patients’ QL after LDR-BT [4–6].

The aim of this study was to describe the relationship between dosimetric parameters and QL outcomes using QL data relating to three years after LDR-BT.

## Materials and methods

### Patients

Between May 2000 and June 2010, we used LDR-BT to treat a consecutive series of 494 patients with LPC (median age 66 years), all of whom had a histopathological diagnosis of prostate cancer based on a needle biopsy. The staging procedures included a digital rectal exam, transrectal ultrasonography, abdominal computed tomography (CT), a bone scan, chest radiography, and PSA determination. No evidence of distant or nodal metastases was required, PSA levels had to be <15 ng/mL, Gleason score ≤7, clinical stage < T3 for implant as monotherapy. The National Comprehensive Cancer Network (NCCN) risk class was low and intermediate risk in 69 and 31 % of the cases, respectively.

The LDR-BT consisted of an I-125 permanent implant with a delivered dose of 145 Gy as exclusive treatment. Thirty-seven percent of the patients underwent short-term pre-implant hormonal therapy, which consisted of three-four months of androgen deprivation therapy for cytoreduction in patients with large prostate volume or obstructive symptoms.

Biochemical relapses were defined on the basis of the Phoenix ASTRO Consensus Conference recommendations [11].

### LDR-BT technique

Patients were implanted with I-125 and pre-loaded needles under fluoroscopy and transrectal ultrasonography. Pre-planning procedures were performed intra-operatively after spinal anesthesia: the prescribed dose to the CTV (prostate + 3 mm in lateral and anterior direction) was 145 Gy and the dosimetric constraints were prostate V100 > 95 %, urethra D10 ≤ 210 Gy and rectal V100 ≤ 0.5 cc. These dosimetric constraints were arbitrarily developed in our Hospital but they strongly correlate with the post planning dosimetry defining the good practice in prostate brachytherapy. In particular, we established V100 ≤ 0.5 cc as dosimetric constraint in ultrasound pre-planning phase and we maintained this cut off value for post-planning assessment.

The post planning evaluation was made using computerized tomography (CT) one month after implantation. Prostate D90 (dose in Gy at 90 % of prostate volume) and V100 (% of prostate volume with ≥145 Gy) of each patient were reviewed and recorded, together with dose volume histograms (DVH) of the organ at risk (OAR), rectum and surrogate urethra based on ultrasound intraoperative images. Patients selection, dosimetric analysis and QL evaluation were defined according to the reference guidelines of the American Brachytherapy Society (ABS) [12, 13].

### QL assessment

#### First study

Until 2004, we used a previously validated patient-filled questionnaire used in a retrospective study of LPC patients treated with external radiotherapy [7]. The questionnaire consisted of 43 items grouped into seven subscales assessing various QL domains: physical well-being (PHY), psychological well-being (PSY), physical autonomy (POW), social relationships (REL), urinary function (URI), rectal function (REC), and sexual function (SEX). Three items explored the patients’ global perception of their QL, and two assessed how the patients perceived the information given to them concerning the disease and its treatment. A standardized score ranging from 0 to 100 was obtained for each subscale: higher non-functional (PHY, PSY, REL, POW) and sexual scale scores indicate better function, whereas higher urinary and rectal scale scores indicate greater impairment.

After they had given their oral consent, the patients were asked to complete the questionnaire one week before and one month after the LDR-BT implantation; the pre-LDR-BT questionnaire was given to each patient by physicians during the first visit and returned on the day of the LDR-BT procedure; the post-LDR-BT questionnaire was given by physicians during the first follow-up visit one month after LDR-BT, and the patients were asked to return it by mail within seven days. In the second phase of the study, we also asked the patients to complete the questionnaire yearly (they were contacted by mail or during the follow-up visit). In an attempt to avoid confounding factors capable of affecting the QL outcomes, we excluded the patients who had experienced a biochemical/clinical relapse or developed a second cancer from the follow-up survey. The patients who refused to complete the follow-up questionnaires were asked to explain the reasons.

#### Second study

The new study started in May 2005, and was designed to assess the different impact of prostatectomy and LDR-BT on QL [8]. The questionnaire was the same as that used in the previous study although, in order to assess the sexual domain in more detail, we added the items suggested by the International Index of Erectile Function-5 (IIEF-5) and UCLA Prostate Cancer Index (UCLA-PCI) that were not included in the previous questionnaire. Other items were also added to make the QL assessment more precise: for example, the new version distinguished stress incontinence from incontinence at rest. The questionnaire consisted of 64 items that were grouped into the seven previous subscales and an additional erection subscale (ERE). As in the first study a standardized score ranging from 0 to 100 was obtained for each subscale: higher non-functional (PHY, PSY, REL, POW) and sexual scale scores indicate better function, whereas higher urinary and rectal scale scores indicate greater impairment.

The new version of the questionnaire was validated in the first 40 patients enrolled in the study, and its validity and reliability was confirmed (data not shown). During the study, the assessment time points for the patients treated with LDR-BT were the same as in the previous study: the yearly assessment was planned for the first five years after implant.

Both questionnaire versions were used only in these studies and were not validated elsewhere. These studies were approved by the local Ethical Committee of Santa Chiara Hospital in Trento.

### Statistical analysis

For the purposes of the present analysis, we considered all of the patients who did not experience disease progression and filled at least three QL questionnaires (pre-LDR-BT, post-LDR-BT, and 1-year questionnaire) as evaluable. Moreover, we assessed QL data until the third year after LDR-BT. The General Linear Model (GLM) for repeated measures was used to compare the scores at the different assessment times, and Bonferroni’s *post hoc* test was used to assess the statistical significance of the differences.

A more than 10 % change in the scores over time constitutes a clinically meaningful difference according to the recommendations of Osoba et al. [14].

The QL outcomes were stratified based on the extent to which the dosimetric constraints of intraoperatively preplanned transrectal ultrasonography had been respected at 1 month post planning CT: D10 urethra dose (≤210 Gy vs >210 Gy), V100 as a percentage of the prostate volume enclosed by the prescription isodose (≤90 % vs 91-95 % *vs* >95 %), and V100 rectal wall volume (≤0.5 cc *vs* >0.5 cc). To avoid the confounding effect of hormonal manipulation of sexuality scales (SEX and ERE), patients who were receiving hormonal treatment before LDR-BT were excluded from the analysis of sexual outcomes. In addition, in assessing the urinary outcomes we stratified the patients according to the baseline urinary function status defined according to URI scale values: poor (a URI score of ≥15; more symptomatic patients) *vs* good (a URI score of <15; less symptomatic patients). This cut off was obtained through a series of questions including frequency of urination, the need to urinate at night, the presence of hematuria, difficulty in initiating urination, urine leakage with effort and at rest, difficulty in holding back urine, jet urine reduced in strength or intensity, pain when urinating. Furthermore, the analyses were separately performed in the patients enrolled in the two studies in order to evaluate potential impact due to the different instrument adopted.

## Results

Data concerning QL outcomes were available from 251 patients (128 from the first study and 123 from the second): all the assessed patients received LDR-BT as exclusive treatment and filled at least pre-LDR-BT, post-LDR-BT, and 1-year questionnaires. One hundred seventy-three patients (69 %) and 97 patients (37 %) were evaluable for 2-year and the 3-year assessment, respectively. Data about all QL outcomes were available in all filled questionnaires.

The median age was 66 years (range 49–77).

### Non-functional scales (PHY, PSY, REL, POW) (Fig. 1)

**Fig. 1:**
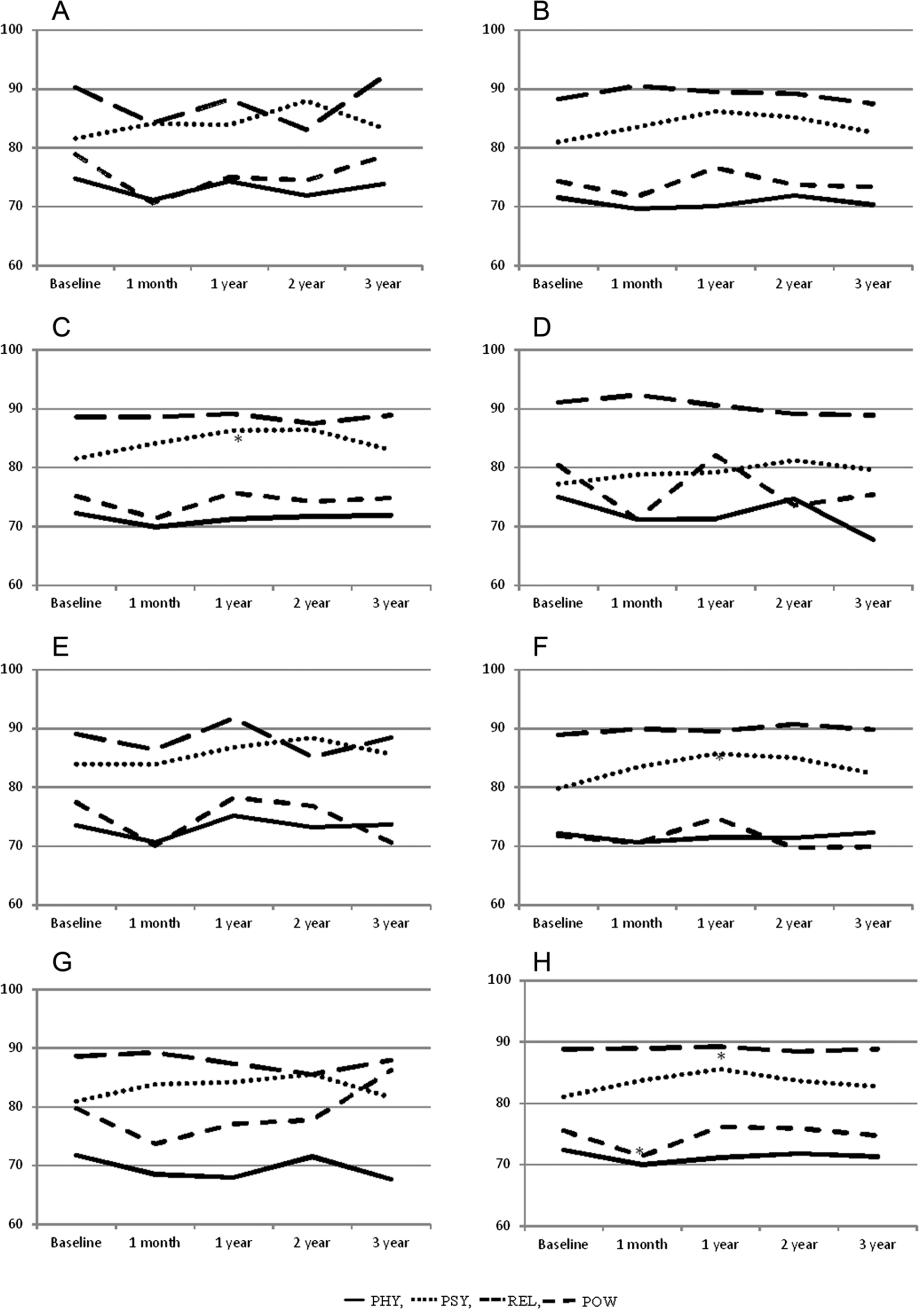
outcomes of non functional scales (median values) where **a**: urethra D10 ≤ 210 Gy, **b**: urethra D10 > 210 Gy, **c**: rectal wall V100 ≤ 0.5 cc, **d**: rectal wall V100 > 0.5 cc, **e**: prostate ≤90 %, **f**: prostate 91-95 %, **g**: prostate >95 %, **h**: results not stratified by the DVH (* p < 0.05)

There was a statistically significant difference in the PHY scale between the pre-implant score and one-month post-implant score (*p* = 0.049), but it was lost in the following three years.

In PSY scale no statistically significant differences from baseline were observed one month post LDR-BT; however we observed an improvement which became significant at one year (*p* = 0.017) and at the following assessment time points, without returning to the baseline level. One year after LDR-BT better PSY scores were observed in patients with V100 prostate volume of 91-95 % (*p* = 0.028) and V100 rectal wall volume ≤ 0.5 cc (*p* = 0.014).

There was no statistically significant difference from baseline in the REL scale after one, two or three years, but the difference was of borderline significance one month post-implantation (*p* = 0.056).

There was no significant difference from baseline in the POW scale one month after LDR-BT or later.

The results were confirmed assessing separately the patients enrolled in the two studies (data not shown).

### Physical function scales (SEX, ERE, URI, REC) (Figs. 2 and 3)

**Fig. 2:**
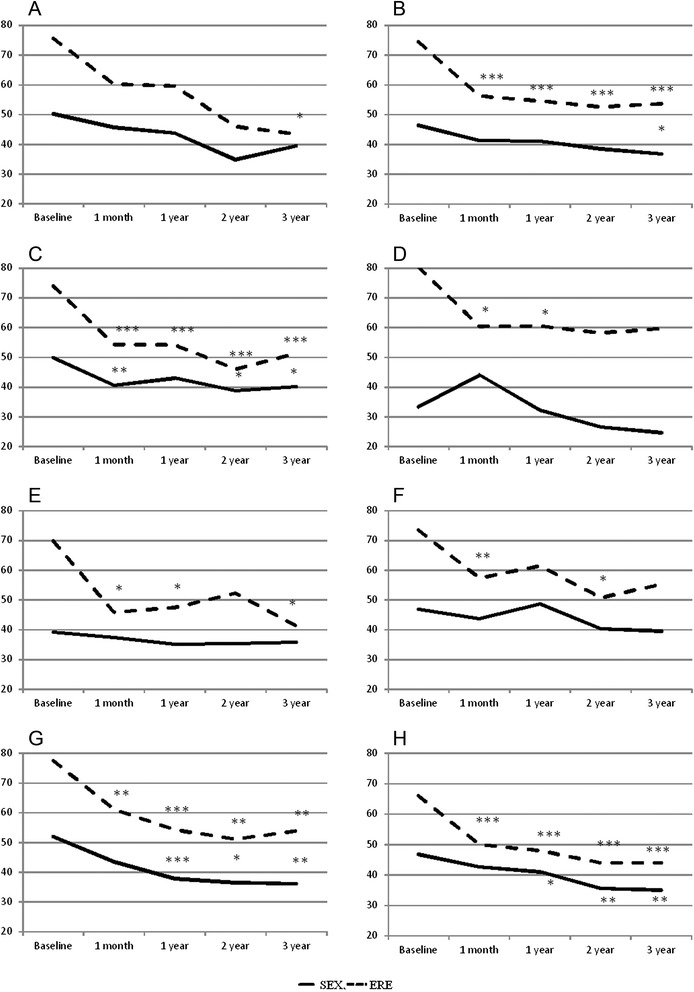
outcomes of sexual function-related scales (median values in patients not receiving hormone therapy) where **a**: urethra D10 ≤ 210 Gy, **b**: urethra D10 > 210 Gy, **c**: rectal wall V100 ≤ 0.5 cc, **d**: rectal wall V100 > 0.5 cc, **e**: prostate ≤90 %, **f**: prostate 91-95 %, **g**: prostate >95 %, **h**: results not stratified by the DVH (* p < 0.05, ** p < 0.001, *** p < 0.0001)

**Fig. 3:**
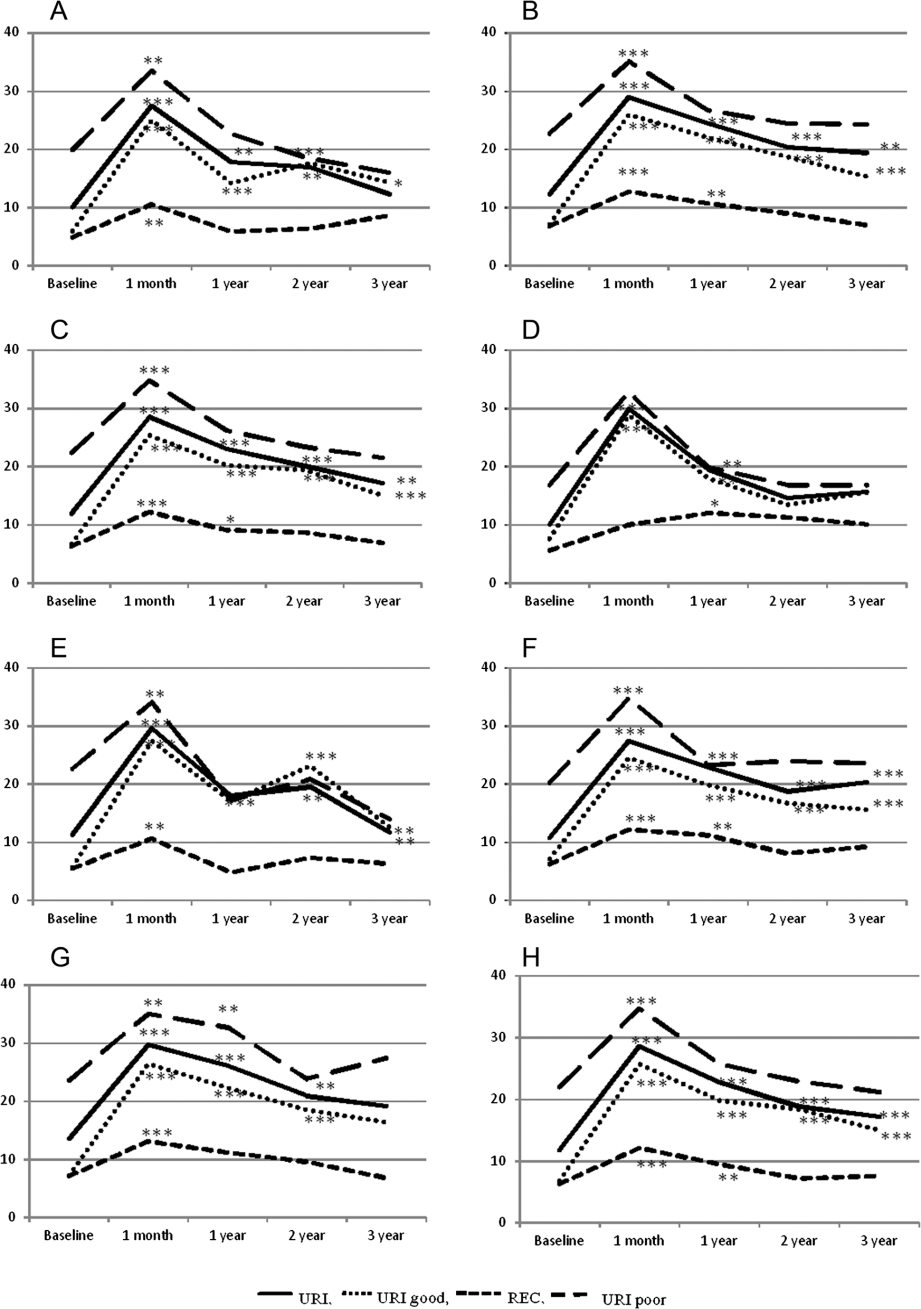
outcomes of other function scales (median values) where **a**: urethra D10 ≤ 210 Gy, **b**: urethra D10 > 210 Gy, **c**: rectal wall V100 ≤ 0.5 cc, **d**: rectal wall V100 > 0.5 cc, **e**: prostate ≤90 %, **f**: prostate 91-95 %, **g**: prostate >95 %, **h**: results not stratified by the DVH (* p < 0.05, ** p < 0.001, *** p < 0.0001)

Progressively impaired sexual function was reported from one month after LDR-BT, despite the differences became statistically significant after two years (*p* = 0.034) and remained significant until the third year (*p* = 0.017). In the subgroup receiving prostate V100 > 95 %, the impairment was always statistically significant from the first year to the third year: *p* = 0.001 after one year, *p* = 0.032 after two years, and *p* = 0.002 after three years. Similarly when V100 rectal wall volume was >0.5 the SEX scale was impaired, despite the greatest effect was observed at the 1-month assessment (*p* = 0.008).

All of the patients reported a clear decrease in the ERE scale after LDR-BT (*p* = 0.0001): this impairment remained significant until the third year (*p* = 0.0001). The greatest impairment was observed in patients with D10 urethra dose >210 Gy, V100 rectal wall volume ≤0.5, and V100 prostate volume >95 %.

The REC scale significantly worsened after LDR-BT (the mean REC score increased from 6.3 to 12.1; *p* = 0.0001), but the difference progressively decreased from the time of the 1-year assessment, and was no longer significantly different from baseline after three years (*p* = 0.367).

Regardless of the statistical significance, the most relevant data concerned post-plan V100 rectal wall volume: when it was ≤0.5 cc (in accordance with the pre-planned constraints), the QL worsened between baseline to one month after LDR-BT (from 6.3 to 12.2), and then progressively improved until it returned to near-baseline levels after three years (the 1-, 2- and 3-years scores were respectively 9.1, 8.7 and 6.9). When V100 rectal wall volume was >0.5, QL worsened between baseline and one month after LDR-BT (from 6.3 to 10.0) and the score was maintained over the three years (Fig 3c, d).

The URI scale significantly worsened immediately after LDR-BT: the mean score increased from 11.8 at baseline to 28.6 one month later, and then gradually improved over the next three years although it always remained significantly different from baseline (the 1-, 2- and 3-year scores were respectively 22.8, 18.8 and 17.2; *p* = 0.0001). The worst impairment was recorded in the patients with good baseline urinary function, whose post-LDR-BT score was about four times higher than the baseline score (25.7 *vs* 6.8) and remained high over time (19.8, 18.4 and 15). The patients with poor baseline urinary function showed a significant worsening in the URI score one month after LDR-BT (34.7 *vs* 22), but this returned to baseline level after two years (22.9) and remained there after three years (21.2). When the post-plan dose at 10 % (D10) of urethra was ≤210 Gy (in accordance with the pre-planned constraint), the QL score worsened between baseline and one month later (from 10.1 to 27.5), but then progressively improved over the years and returned to near-baseline levels after three years (1-, 2- and 3-year scores of respectively 17.8, 16.9 and 12.3). When D10 urethra was ≥210 Gy, the QL score worsened between baseline and one month (from 10.1 to 29.0) and never returned to baseline level (Fig. 3a, b). The results were confirmed assessing separately the patients enrolled in the two studies (data not shown).

## Conclusions

To the best of our knowledge, this is the first study assessing dosimetric parameters and their impact on all QL domains after LDR-BT, and the results show a substantial impact on functional scales and a marginal effect on non-functional scales.

Most of the previously published studies focused on treatment side effects but did not comprehensively assess the impact on the patients’ feelings or QL dimensions. A recent study from Hannover University described a series of 186 consecutive patients, whose morbidity after I-125 brachytherapy was prospectively assessed using the International Prostate Symptom Score (IPSS) and the IIEF-5 over a median follow-up of 30 months. All of the scores troughed after six weeks and normalized after 24 months. The correlation between segmental dosimetry and the scores at the different time points demonstrated that only the prostate base scores remained statistically significant in multivariate regression analyses at all time intervals (p <0.00) [15].

Allen et al. evaluated the relationship between urinary morbidity after LDR-BT and urethral doses at the baseline, mid-prostate, apical and urogenital diaphragm. The study did not find any statistically significant influence on IPSS at univariate or multivariate analysis [16].

Thomas et al. found that radiation dose at the urethral base, larger prostate ultrasound planning volumes and needle number predicted increased urinary toxicity following LDR-BT [17].

Vordermark et al. reported QL data in 74 low-risk prostate cancer patients treated with permanent I-125 brachytherapy. The study demonstrated that both urinary and bowel symptoms strongly correlated with pre-treatment scores during the first year after the treatment, suggesting the importance of screening patients for baseline symptoms for treatment choice [6].

The most striking results of our study concerned physical function, particularly the rectal and urinary scales.

As expected, a progressive impairment in sexual and erectile function was reported one month after LDR-BT, and remained statistically significant after the third year. Irradiation of the penile bulb is a considerable issue because this organ is considered at risk for erectile damage [18]. As this dosimetric parameter was not included in the pre-operative planning evaluation, the lack of data is a limitation in our study.

Rectal function significantly worsened after LDR-BT, but gradually improved after the 1-year assessment and was no longer significantly different from baseline after three years (*p* = 0.367).

Regardless of statistical significance, the QL depends on V100 rectal wall volume irradiation (≤ or > 0.5 cc). When the percentage of rectal volume receiving ≥145 Gy was ≤0.5 cc, the QL worsened between baseline and one month after LDR-BT, but progressively improved over the years, and returned to baseline levels after three years; when rectal V100 was > 0.5, the QL worsened between baseline and one month, and never improved again (Fig. 3c, d).

Urinary function significantly worsened immediately after LDR-BT, but gradually improved over the next three years, although the mean URI score was always significantly different from baseline. The subgroup analysis showed that the patients with good baseline urinary function experienced less improvement at long term follow up than those with poor baseline urinary function: the former had a post-LDR-BT URI score that was about four times higher than baseline (25.7 *vs* 6.8) and remained higher over time, whereas the latter showed a significant worsening one month after LDR-BT (34.7 *vs* 22), but returned to baseline level after two years (22.9) and maintained this level after three years (21.2). Based on these findings, it seems that brachytherapy can also be proposed to patients with a suboptimal urinary function.

The most interesting data concern the D10 urethra parameter: when it was ≤210 Gy, the QL worsened between baseline and one month after LDR-BT, but then progressively improved and returned to near-baseline levels after three years; when it was >210 Gy, the QL worsened between baseline and one month later, and never returned to baseline levels (Fig. 3a, b).

The outcomes relating to the non-functional scales (PHY, PSY, REL, POW) were usually not statistically significant. The results are difficult to interpret, but suggest that LDR-BT has only marginal effects on LPC patients’ QL.

Many studies have considered the relationships between dosimetric parameters and biochemical outcomes or treatment side effects after LDR-BT, but ours is the first to investigate the impact of post-planning dose volume histograms on the QL. It provides further evidence that a good implant and respect for the usual planning constraints on the rectum and urethra are important factors for maintaining a good QL degree.

## Abbreviations

QL: Quality of life
LPC: Low-risk prostate cancer
LDR-BT: Low-dose-rate brachytherapy
PHY: Physical
PSY: Psychological
POW: Physical autonomy
REL: Relationships
URI: Urinary
REC: Rectal
SEX: Sexual
CT: Computed tomography
PSA: Prostate specific antigen
NCCN: National Comprehensive Cancer Network
DVH: Dose volume histograms
OAR: Organ at risk
BT: Brachytherapy
IIEF-5: International Index of Erectile Function-5
UCLA-PCI: UCLA Prostate Cancer Index
GLM: General Linear Model
